# Building the crops of tomorrow: advantages of symbiont-based approaches to improving abiotic stress tolerance

**DOI:** 10.3389/fmicb.2014.00283

**Published:** 2014-06-06

**Authors:** Devin Coleman-Derr, Susannah G. Tringe

**Affiliations:** Joint Genome Institute, Walnut CreekCA, USA

**Keywords:** symbiosis, abiotic stress, agriculture, plant growth promotion, plant–microbe interactions, drought

## Abstract

The exponential growth in world population is feeding a steadily increasing global need for arable farmland, a resource that is already in high demand. This trend has led to increased farming on subprime arid and semi-arid lands, where limited availability of water and a host of environmental stresses often severely reduce crop productivity. The conventional approach to mitigating the abiotic stresses associated with arid climes is to breed for stress-tolerant cultivars, a time and labor intensive venture that often neglects the complex ecological context of the soil environment in which the crop is grown. In recent years, studies have attempted to identify microbial symbionts capable of conferring the same stress-tolerance to their plant hosts, and new developments in genomic technologies have greatly facilitated such research. Here, we highlight many of the advantages of these symbiont-based approaches and argue in favor of the broader recognition of crop species as ecological niches for a diverse community of microorganisms that function in concert with their plant hosts and each other to thrive under fluctuating environmental conditions.

## INTRODUCTION

Climate change and an increasing world population are predicted to drastically increase the global need for arable farmland, a resource that is already in high demand (Barrow et al., 2008). With the world population expected to reach 9 billion by 2050, it is estimated that the global food supply will need to increase by 70% to meet rapidly rising demand (Editorial, 2010). Changes in the global climate may well compound this challenge, as predicted increases in drought and temperature-related stresses are expected to reduce crop productivity (Ciais et al., 2005; Grover et al., 2010; Larson, 2013).

This large expansion in agricultural output will require both improvements in crop yield as well as the cultivation of additional farmland. One direct effect of this trend will be the steadily increasing prevalence of farming on marginal, arid, and semi-arid lands, especially in the developing world (Lantican et al., 2003; Köberl et al., 2011). Even without considering the effects of climate change, semi-arid, and arid lands often present a host of abiotic challenges to plant growth, including extreme temperatures, excess radiation, and poor nutrient and water availability (Yang et al., 2009).

The historical approach to mitigate the negative effects of abiotic stresses on crop yield has been the creation of stress-tolerant cultivars (Barrow et al., 2008; Eisenstein, 2013). Conventional breeding techniques have enabled the development of crop varietals with increased yields and greater tolerance to a variety of abiotic stresses (Atkinson and Urwin, 2012), but are both time and labor intensive; genetic engineering of crops with improved stress tolerance is faster, but comes with its own set of drawbacks. Furthermore, both methods often neglect the complex ecological context of the soil environment in which the crop is grown (Morrissey et al., 2004).

In recent years, plant-associated microbial communities have received considerable attention for their ability to confer many of the same benefits to crop productivity and stress resistance as have been achieved through plant breeding programs (Mayak et al., 2004; Barrow et al., 2008; Marulanda et al., 2009; Tank and Saraf, 2010; Marasco et al., 2012). It is now well recognized that all plants, and nearly all tissues within the plant, are inhabited by a variety of microorganisms (Partida-Martínez andHeil, 2011; Berg et al., 2013), many of which offer benefits to the host, improving nutrient uptake, preventing pathogen attack, and increasing plant growth under adverse environmental conditions (Yang et al., 2009; Turner et al., 2013). In return these microorganisms receive shelter from the surrounding environment and access to a carbon-rich food supply. The most well-studied of these symbionts include the mycorrhizal fungi, which enhance nutrients uptake (Bonfante and Anca, 2009) and root-nodulating bacteria, which fix nitrogen from the surrounding soil (Lugtenberg and Kamilova, 2009), but many other novel plant growth-promoting microorganisms (PGPM) continue to be identified each year. These organisms confer stress resistance via diverse mechanisms recently reviewed elsewhere (Lugtenberg and Kamilova, 2009; Yang et al., 2009; Grover et al., 2010; de Zelicourt et al., 2013; Nadeem et al., 2014). Importantly, efforts are being made to harness these naturally occurring, soil-derived beneficial microbes for large-scale improvement of crop performance in agriculture (Nadeem et al., 2014).

In this article, we will highlight some of the advantages associated with symbiont-based approaches to increasing crop resistance to abiotic stress, with a focus on engineering increased tolerance to drought, which is the most critical and prevalent factor for crop production in many parts of the world (Castiglioni et al., 2008; Grayson, 2013). We present suggestions for future directions of abiotic stress tolerance improvement in crop plants, including the use of cutting edge genomic technologies for the identification and selection of candidate symbionts and the functional modules they employ for enhancing host growth, as well as an assessment of current agronomic practices in the light of modern understanding of microbial community influence over plant phenotype. We conclude with an argument in favor of increased collaboration between conventional breeding programs and microbial-based research for crop improvement and, more generally, for a broader conceptual understanding of crop productivity as a complex product of plant genetics and microbial community function.

## LIMITATIONS ASSOCIATED WITH DIRECT ENGINEERING OF INCREASED STRESS TOLERANCE INTO CROP PLANTS

The success of plant biotechnology programs has helped the world’s food supply keep pace with the increasing rate of population growth (Morrissey et al., 2004). Novel crop varietals, with superior yields as well as increased tolerance to biotic and abiotic stresses, have been continuously produced for decades through conventional plant breeding programs, and more recently through genetic engineering (Atkinson and Urwin, 2012). Despite the undeniable success of these past efforts and their continued applicability to drought-tolerance in crop species, each of these methods has its drawbacks, which should be fully considered. Plant breeding is highly time consuming, as well as labor and cost intensive (Ashraf, 2010; Eisenstein, 2013). Additionally, in the quest for the improvement of a particular trait, such as drought tolerance, certain (often unknown) desirable traits can be unintentionally lost from the host’s gene pool during conventional breeding (Philippot et al., 2013). Perhaps the largest drawback, however, is that plant breeding only confers benefit to a single host species, and this benefit is often not easily transferable to other crop systems, as the genetic components responsible for the improvements frequently remain unidentified.

To avoid the time and labor costs associated with conventional breeding, some researchers have turned to generation of transgenic lines for producing varietals with improved plant growth regulators, antioxidants, organic osmolytes or other factors capable of increasing drought tolerance (Eisenstein, 2013). Unfortunately, the vast majority of these are developed and tested in the greenhouse, rather than in the field and claims made regarding their performance are often inflated compared to actual results in agricultural settings, due to the large array of abiotic and biotic factors left out of the initial experiments (Ashraf, 2010). Additionally, these transgenic crops often must pass rigorous food and environmental safety regulations and trials before becoming marketable, which adds additional time to the product development process (Eisenstein, 2013). Furthermore, release of a transgenic product into the marketplace does not guarantee its success, as public response to use of genetically modified crops varies considerably from country to country (Fedoroff et al., 2010).

Both the conventional breeding and genetic engineering based approaches may rely too heavily on the assumption that plants function as autonomous organisms regulated solely by their genetic code and cellular physiology (Barrow et al., 2008), although plant–microbe interactions can heavily influence crop response to environmental conditions. Many field trials of new stress-tolerant cultivars simply have not addressed microbial influence on improved performance (Budak et al., 2013; Swamy and Kumar, 2013; Cooper et al., 2014). Greenhouse trials are often conducted with standard sterilized potting soils and sterilized soil amendments (Porch, 2006; Waterer et al., 2010; Witt et al., 2012) in an attempt to create a microbe-free growth environment, an artificial context rarely if ever found in nature (Friesen et al., 2011; Partida-Martínez and Heil, 2011). By doing so, they not only neglect one of the top determinants of phenotypic output, they may also miss vertically transmitted symbionts present within the plant seed (Barrow et al., 2008), which could lead to overestimations of the effect of host genotype on plant phenotype.

## ADVANTAGES OF SYMBIONT-BASED APPROACHES TO IMPROVING STRESS TOLERANCE

**FIGURE 1:**
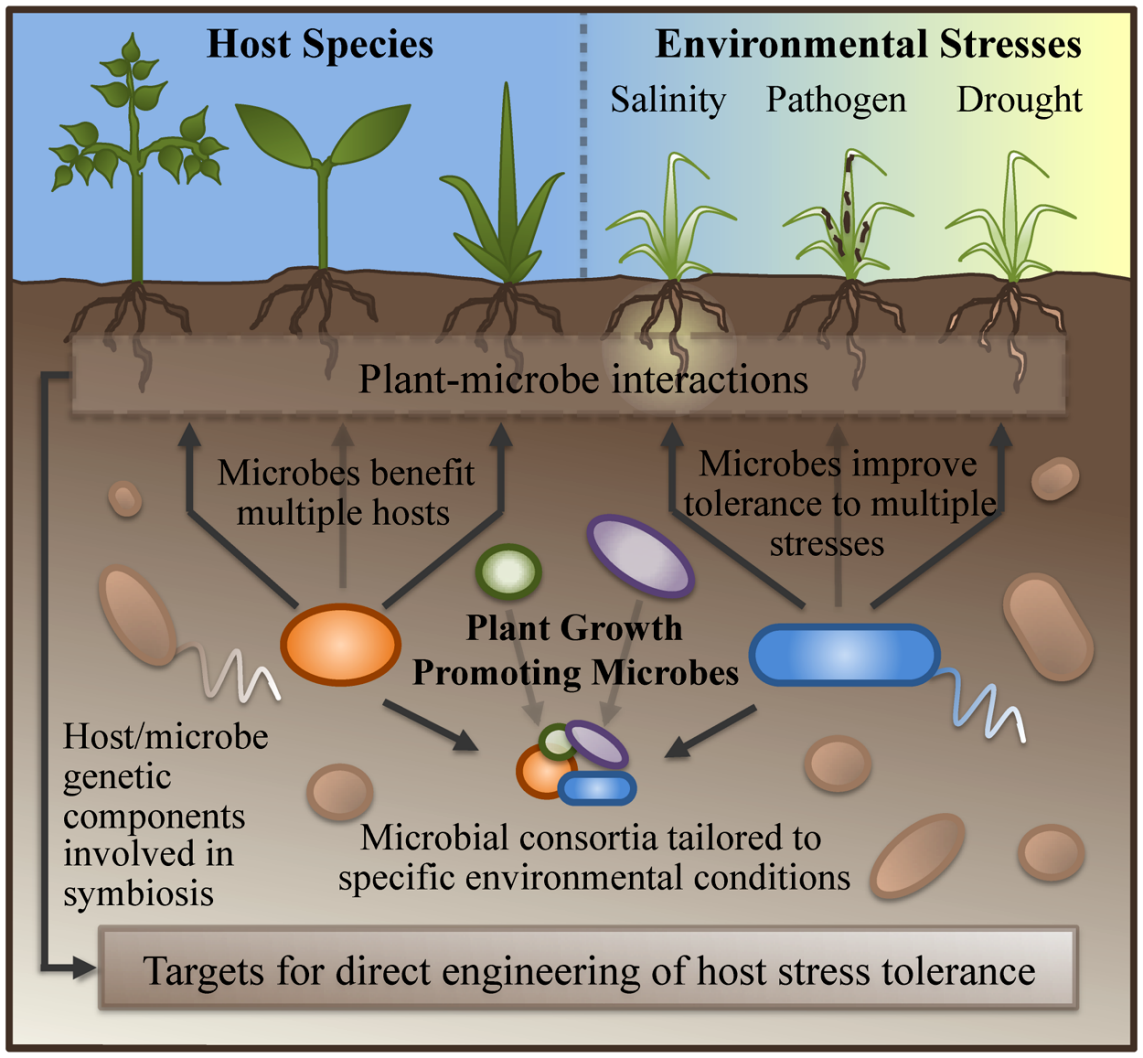
**Advantages of symbiont-based approaches to improving stress tolerance in crops.** Plant-growth promoting microbes are capable of conferring benefits to multiple species of plant hosts, and of offering improved tolerance to multiple stresses simultaneously. Inoculations with combinations of PGPM can be tailored to specific environmental conditions. Dissection of plant–microbe interactions during symbiosis has the potential to reveal both the microbial and host genetic components responsible for improved stress tolerance; these may serve as targets for plant-breeding/genetic-engineering based approaches to improving stress tolerance in the host.

Compared with methods for directly engineering stress tolerance into the host described above, symbiont-based approaches to improving stress tolerance offer some clear advantages (**Figure 1**). First, microbial symbionts are frequently capable of conferring stress tolerance to a wide variety of diverse plant hosts, and many PGPM can confer benefits to both monocots and dicot crop species (Timmusk and Wagner, 1999; Redman et al., 2002; Zhang et al., 2008). The bacterium *Achromobacter piechaudii*, isolated from dry riverbeds of southern Israel, was capable of increasing salt and drought resistance in both pepper and tomato (Mayak et al., 2004). Using olive trees, tomato, grapevine, and pepper plants, Marasco et al. (2013) have demonstrated that microbes isolated from the roots of one host species cultivated under desert farming conditions are capable of improving the growth of a different host species when grown under a water-stress regime. The ability to transfer stress-resistance solutions from one crop species to another through a microbial inoculum has the potential to save years of plant breeding effort.

Secondly, PGPM frequently confer more than one type of abiotic and/or biotic stress tolerance (Mayak et al., 2004; Rodriguez et al., 2008), and crops grown on arid and semi-arid lands typically suffer from multiple stress factors. It has been shown that *Arabidopsis* plants in symbiosis with *Paenibacillus polymyxa* have increased drought tolerance as well as improved resistance to pathogen attack (Timmusk and Wagner, 1999). Waller et al. (2005) demonstrated that barley plants inoculated with the fungus *Piriformospora indica* have both increased resistance to *Fusarium* and *Blumeria* infections and increased salt tolerance. These examples of microbes conferring multiple benefits are likely due to the fact that many symbionts exert their influence over the plant host through manipulating plant hormone pathways (Glick et al., 2007; Friesen et al., 2011) and that considerable cross-talk exists between plant stress response pathways (Atkinson and Urwin, 2012).

Thirdly, plant-associated microbial species represent a vast reservoir of genetic information that has coevolved with their hosts under natural environmental conditions. These microbes can add genetic flexibility to the adaptation of comparatively sessile and longer-lived plants (Barrow et al., 2008). The concept of “habitat-specific symbioses,” put forth by Rodriguez et al. (2008), is one of the most intriguing discoveries pertaining to microbial contributions to stress tolerance made in recent years. Their research found that salt, drought, and disease resistance were each individually conferred by specific fungal symbionts that had been harvested from coastal, arid, and agricultural environments, respectively. Furthermore, they found that these beneficial effects could be conferred on different plant host species, including both monocots and dicots. These insights suggest that the foundation for the growth-promoting effects of microbial symbionts is based on the co-evolution of the association between plant and microbe under adverse environmental conditions (Rodriguez et al., 2008). For the purposes of developing novel biotechnological agents for use in agriculture, this study supports the idea that the optimal place to look for PGPM that confer resistance to a specific environmental stress is in soils where that stress is a regular phenomenon.

## FUTURE DIRECTIONS OF ABIOTIC STRESS TOLERANCE IMPROVEMENT IN CROP PLANTS

Microbial species with plant-growth promoting capabilities are both numerous and easier to characterize now than ever before. A considerable fraction of endophytes isolated from crops appear to have measurable effects on host fitness (Friesen et al., 2011). Two recent studies found that more than 25% of bacteria isolated from cultivated crops had plant growth promoting activities (Hassan et al., 2010; Marasco et al., 2012). While the identification of microbial endophytes has been challenging in the past due to the frequent lack of plant–host symptoms, localized colonization, intimate integration with plant cellular structures, and lack of cultivability, recent advances in genomic technologies have helped make this process faster and cheaper (Berg et al., 2013). A recent technique for selective depletion of chloroplast and mitochondrial-derived 16S amplicons allows for vastly increased resolution of bacterial endophyte populations derived from within plant tissues (Lundberg et al., 2013). While in the past whole-genome sequencing of candidate symbionts was only possible for cultivable species, it is now possible to obtain draft genomes of microbial endophytes in a high-throughput fashion using single-cell sorting coupled with next-generation sequencing technologies (Woyke et al., 2006). Understanding the genomic content of these PGPMs will enable us to better understand the mechanisms behind the conferred stress-tolerances, as well as cultivate them for experimental investigation (Pope et al., 2011).

As more and more genomes from PGPM become available, our ability to identify the shared genetic components or metabolites that are responsible for conferring specific abiotic stress advantages increases. Through a transcriptomic analysis of the symbiosis between oilseed rape and *Stenotrophomonas rhizophila*, a recent study identified spermidine as a novel PGPM regulator of plant abiotic stress (Alavi et al., 2013). Identification of the genetic components within PGPMs that are responsible for alleviating abiotic stress may in some cases yield potential targets for transgenic modification of the host organism (Nadeem et al., 2014). Recently, bacterial cold-shock proteins transformed into various plant species led to increased tolerance to a variety of abiotic stresses, including cold, heat, and drought (Castiglioni et al., 2008).

Investigation of the mechanisms by which PGPM confer stress-tolerance to their plant hosts is another avenue for identifying targets for direct transgenic manipulation of stress response in crops. Recent technological advances in cell-type specific transcriptomics (Taylor-Teeples et al., 2011), combined with an experimental system designed to examine host transcription during symbiosis with PGPM, could allow for a precise dissection of the genetic signaling mechanisms responsible for increased stress tolerance. An improved understanding of these host mechanisms could provide potential candidate loci for transgenic or plant-breeding strategies aimed at plant–host improvement (Grover et al., 2010). For example, salt tolerance induced by *Bacillus subtilus* was shown to be the result of tissue specific modulation of the expression of the *Arabidopsis* Na^+^/K^+^ transporter, *HKT1* (Zhang et al., 2008). Similarly, drought resistance in *Arabidopsis* as a result of inoculation with *P. polymyxa* was related to strong upregulation of the host gene *ERD15* (Timmusk and Wagner, 1999).

Finally, there is a need for rethinking modern agronomic practices in light of our current understanding of the importance of host-associated microbial communities for plant productivity and health. Current large-scale agricultural systems rely heavily on monoculture cropping systems, in many cases without between-season crop rotation, which has been shown to lead to the build up of specialized plant pathogens, increased disease incidence, and decreased yield (Berendsen et al., 2012; Gentry et al., 2013). Research is being conducted to determine if the use of specific cover crops can be used to promote and maintain a beneficial microbiome between growing seasons for important crop species (East, 2013). Current methods of tilling may also negatively impact the plant microbial community; alternatives, including “conservation-” or “zero-tillage,” may have the potential to promote a healthy belowground microbiome by reducing moisture loss and maintaining naturally occurring strata within the soil, which helps support microbial biodiversity (East, 2013).

## CONCLUSION

As with the plant-breeding and transgenic approaches to engineering stress-resistance in tomorrow’s crops, there are of course challenges associated with symbiont based strategies that will need to be overcome. One potential challenge will be detangling synergistic and antagonistic effects of different microorganisms within the plant microbiome (Trabelsi and Mhamdi, 2013). Research has demonstrated synergistic effects of multiple PGPM (Figueiredo et al., 2008), and another study has identified a virus present within a plant growth promoting fungus as the causative agent of heat resistance conferred to a tropical grass (Márquez et al., 2007). A second challenge stems from the fact that while many PGPM have been shown to confer their benefits across multiple host species, it is clear that this is not always the case. In some studies, the host species (and even host cultivar) has been shown to play a significant role in driving microbial community composition and activity (Ofek et al., 2013; Philippot et al., 2013), selecting for and against particular microbial partners. Additionally, interactions between the PGPM and the members of the existing microbial community could alter or negate the potential beneficial effects of the microbe (Schippers et al., 1987). Due to the complexity of interactions among the microbes, host, and environment, there is the potential that a PGPM that confers benefit in one context may have a null, or even negative, effect in a different context; therefore, considerable work will need to be done to determine the range of applicability for each PGPM as a beneficial agricultural agent. A third challenge, which is equally important for both symbiont and host-based methods of improving stress tolerance, will be unraveling the complex relationships between the various biotic and abiotic stress responses. Research programs aimed at developing tolerance to a particular stress do not necessarily test susceptibility to other stresses; due to the intrinsically related nature of the pathways governing stress response, later field trials have in some instances revealed increased susceptibility to other stresses (Atkinson and Urwin, 2012). Lastly, methods of microbial delivery within field settings and stable integration of PGPMs into the agricultural soil ecosystem will need improvement. While many applications of PGPMs to crops in field settings have demonstrated significant improvements to stress tolerance (Celebi et al., 2010; Mengual et al., 2014; Rolli et al., 2014), others have shown inconsistent or even negative effects (Nadeem et al., 2014). One promising method of stabilizing beneficial effects of PGPM in the field involves the inoculation of a microbial consortium of PGPM, as opposed to a single PGPM species. Combining PGPM known to grow and perform well together will likely increase the resilience of the inoculum and its beneficial effects, and additionally allow for tailoring the community to respond to specific combinations of abiotic and biotic stresses (Trabelsi and Mhamdi, 2013).

Agriculture currently accounts for 70% of human fresh water use, and in many parts of the world this rate of water consumption exceeds local regeneration rates, leading to unsustainable reliance on underground aquifers that are rapidly depleting (Castiglioni et al., 2008; Jiao, 2010). Given this, it is not surprising that drought and other water-related stresses are considered by many to be the most significant threats to global agricultural security in the near future. Encouragingly, in the research conducted by Rodriguez et al., the “habitat-specific symbionts” selected from a coastal site, a geothermal site, and an agricultural site shared one trait: the ability to confer drought resistance. Rodriguez et al. (2008) hypothesize that the ability of fungal endophytes to confer drought tolerance may be a common evolutionary relic from when plants left the ocean, as fungal symbiosis is thought to be in part responsible for the movement of plants to land. If this turns out to be the case, proponents of symbiont-based approaches to increasing stress resistance in crop plants may do well to focus their efforts on drought and other water-related stresses.

In the future, there is a need for more collaboration between the host-focused and symbiont-focused approaches to mitigating abiotic stress in crop plants. Medical science has in recent years undergone a profound restructuring of its understanding of the microbiome housed within the body and its impact on human health (East, 2013). There is a clear parallel here for plant science, with implications that have the potential to change the face of agriculture and help us to meet the challenges confronting humanity in light of our expanding population and changing planet. The fundamental change required is a broader recognition that plants do not exist as autonomous organisms governed entirely by their genetic blueprints, but rather serve as ecological niches for diverse communities of easily overlooked microbes, which work in concert with the plant to survive in a wide range of stressful environmental conditions.

## Conflict of Interest Statement

The authors declare that the research was conducted in the absence of any commercial or financial relationships that could be construed as a potential conflict of interest.
